# Molecular mechanisms mediating asymmetric subcellular localisation of the core planar polarity pathway proteins

**DOI:** 10.1042/BST20190404

**Published:** 2020-08-21

**Authors:** Carl Harrison, Hongyu Shao, Helen Strutt, David Strutt

**Affiliations:** Department of Biomedical Science, University of Sheffield, Western Bank, Sheffield S10 2TN, U.K.

**Keywords:** PCP, phosphorylation, planar cell polarity, planar polarity, post-translational modification, ubiquitination

## Abstract

Planar polarity refers to cellular polarity in an orthogonal plane to apicobasal polarity, and is seen across scales from molecular distributions of proteins to tissue patterning. In many contexts it is regulated by the evolutionarily conserved ‘core' planar polarity pathway that is essential for normal organismal development. Core planar polarity pathway components form asymmetric intercellular complexes that communicate polarity between neighbouring cells and direct polarised cell behaviours and the formation of polarised structures. The core planar polarity pathway consists of six structurally different proteins. In the fruitfly *Drosophila melanogaster*, where the pathway is best characterised, an intercellular homodimer of the seven-pass transmembrane protein Flamingo interacts on one side of the cell junction with the seven-pass transmembrane protein Frizzled, and on the other side with the four-pass transmembrane protein Strabismus. The cytoplasmic proteins Diego and Dishevelled are co-localised with Frizzled, and Prickle co-localises with Strabismus. Between these six components there are myriad possible molecular interactions, which could stabilise or destabilise the intercellular complexes and lead to their sorting into polarised distributions within cells. Post-translational modifications are key regulators of molecular interactions between proteins. Several post-translational modifications of core proteins have been reported to be of functional significance, in particular phosphorylation and ubiquitination. In this review, we discuss the molecular control of planar polarity and the molecular ecology of the core planar polarity intercellular complexes. Furthermore, we highlight the importance of understanding the spatial control of post-translational modifications in the establishment of planar polarity.

## Introduction

Many cells require asymmetrical architecture — termed polarity — that allows structures to be compartmentalised within a cell in order to fulfil their function. Apicobasal polarity is common in many cell types such as neurons, where it enables unidirectional signal transduction, and epithelial cells, enabling unidirectional secretion. Cells can also be polarised in an orthogonal plane, which is termed planar polarity, also known as planar cell polarity (PCP). Planar polarity allows the placement of structures to be co-ordinated with the tissue axis and with neighbouring cells. Planar polarity is evolutionarily conserved [1] and has been reported in a range of tissues in both vertebrates and invertebrates, and is vital to proper tissue development and function. Examples of planar polarity enabled processes include cilia mediated convection currents in the oviduct, the arrangement of hair cells in the inner ear, and the overlapping nature of mammalian fur (reviewed in [2–4]). Planar polarity has been reported from as early as epiblast formation in zebrafish [5] and primitive endoderm formation in mouse [6]. Its loss is implicated in neural tube closure defects [7], spermatogenesis defects [8], deafness [9], and heart defects [10]. As such the underlying mechanisms are of broad interest.

**Figure 1.**
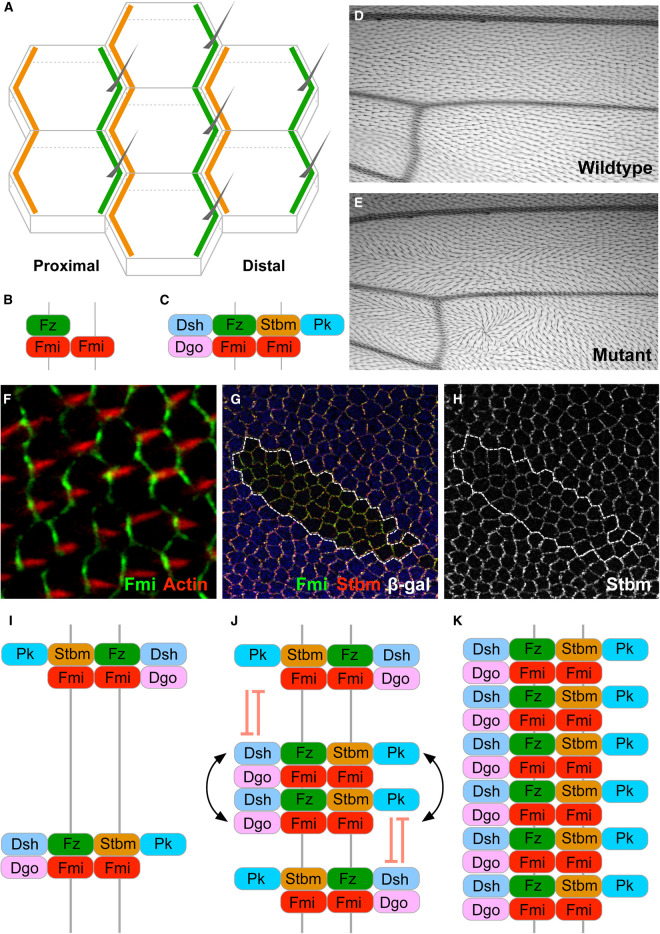
Principles of planar cell polarity. (**A**) Schematic of localisation of core planar polarity pathway protein complexes in cells of the *Drosophila* pupal wing. Proximal localisation of Stbm (orange) and distal localisation of Fz (green) at apicolateral cell junctions leads to trichome (grey) emergence from distal cell edges. (**B** and **C**) Evolution of symmetry breaking cluster formation. (**B**) Fmi homodimers are stabilised by Fz localisation on one side of the complex at junctions between neighbouring cells. (**C**) Stbm localises to the apposing cell edge and the cytoplasmic proteins Pk, Dsh and Dgo localise proximally and distally in the complex as shown. (**D**) Image of dorsal surface of a wild-type *Drosophila* wing, showing uniform distal orientation of trichomes. Proximal is left, and anterior is up. (**E**) Image of dorsal surface of wing from a *pk^pk-sple13^* mutant fly, showing a swirled trichome pattern. (**F**) Confocal microscope image of *Drosophila* pupal wing epithelium, immunolabelled for Fmi (green, localised preferentially to proximodistal cell boundaries) and showing actin-rich trichomes (red, emerging from distal cell edges). (**G** and **H**) Confocal microscope image of a *Drosophila* pupal wing epithelium genetically mosaic for *dsh^V26^* mutant tissue, marked by loss of blue β-gal immunolabelling and outlined in white (**G**). Fmi (green) and Stbm (red, or white in **H**) are asymmetrically localised at proximodistal cell boundaries at the apicolateral cell junctions (left and right cell edges) in wild-type tissue, but lose this asymmetric localisation in mutant tissue. This shows that Dsh activity is required for planar polarisation of core pathway components such as Fmi and Stbm. (**I**–**K**) Molecularly asymmetric complexes (**I**) are sorted by feedback interactions (**J**) so that they all align in the same orientation (**K**). Complexes of opposite orientation are destabilised (red inhibitory symbols) and complexes of the same orientation are stabilised (black arrows).

At a molecular level, planar polarity principally involves the formation of intercellular complexes with heterologous components. Planar polarity can be genetically regulated by two well-characterised pathways: the Fat-Dachsous (Ft-Ds) pathway and the core planar polarity pathway (‘core pathway', see Table 1). In the Ft-Ds pathway, the atypical cadherins Ft and Ds localise to opposite cell ends [11–13], where they interact intercellularly to form a heterophilic complex [14,15]. The *Drosophila* core pathway protein distribution consists of an intercellular homodimer of the atypical cadherin Flamingo (Fmi; also known as Starry Night [Stan]) [16] and asymmetric localisation of associated proteins (summarised in Figure 1A,C). The seven-pass transmembrane protein Frizzled (Fz) and the cytoplasmic proteins Dishevelled (Dsh) and Diego (Dgo) are found on one side of the Fmi homodimer [17–21], and on the other side are the four-pass transmembrane protein Strabismus (Stbm, also known as Van Gogh [Vang]) and the cytoplasmic protein Prickle (Pk) [22,23].

**Table 1:** Planar polarity proteins in flies and vertebrates

Drosophila protein	Symbol	Vertebrate homologues
*Ft-Ds pathway*		
Fat	Ft	Fat
Dachsous	Ds	Dchs
Four-jointed	Fj	Fjx1
*Core pathway*		
Flamingo/Starry Night	Fmi/Stan	Celsr
Frizzled	Fz	Fz/Fzd
Strabismus/Van Gogh	Stbm/Vang	Vangl
Prickle	Pk	Pk
Dishevelled	Dsh	Dvl
Diego	Dgo	Diversin

Due to simple husbandry, a small genome and fast reproductive cycle, the fruitfly *Drosophila melanogaster* has been at the forefront of genetic research for over a century. Readily accessible readouts of planar polarity in *Drosophila* include photoreceptor orientation within the eye (ommatidia), the organisation of surface bristles on the body, and wing hair (trichome) placement originating from distal cell edges (Figure 1A,D–F). *Drosophila* genetic tools allow for powerful and versatile experimental design. These include the rapid generation of transgenic animals, the ability to create mosaic mutants (Figure 1G,H) and the ability to express genes ectopically. These experimental paradigms have allowed for the robust assessment of the roles of particular gene products during development, including the elucidation of the genetic control of planar polarity. Here we primarily discuss mechanisms of planar polarity establishment in the developing *Drosophila* wing, which is a simple epithelial sheet, allowing easy imaging of fixed samples (Figure 1F–H), while also being accessible for live imaging.

## Tissue level control of patterning in planar polarity

A pervading question within the planar polarity field regards the nature of a global cue to co-ordinate polarity with the tissue axis. Does it exist? And if so, what is it? Structures within epithelial sheets that are hundreds of cells wide are all co-ordinated to be planar polarised on parallel vectors. Global polarity of the Ft-Ds system appears to be based in part upon a vectoral gradient of Four-jointed (Fj) distribution, which is expressed in a graded fashion along the proximodistal axis of the developing wing [24,25]. Both Ft and Ds are phosphorylated by the kinase activity of Fj, with opposing effects on their heterophilic binding affinities. This results in phosphorylated Ft having an increased binding affinity for Ds, but phosphorylated Ds having a decreased affinity for binding Ft [26–28]. This leads to a local imbalance of Ft and Ds localisation within each cell, which in turn propagates to neighbouring cells. A 3% difference in Fj activity between neighbouring cells was found to be sufficient to generate Ft-Ds planar polarity *in silico* [28].

A global cue for the core pathway has been more elusive. Secreted Wnt ligands are known to bind to Fz receptors [29], and Wnts are expressed in gradients in the developing *Drosophila* wing [30–32]. Ectopic overexpression of Wnts can reorient trichomes in the wing [33]. However, an instructive role for Wnts in directing trichome orientation has been difficult to demonstrate using loss-of-function mutations (reviewed in [34]). In addition, it has been proposed that the Ft-Ds system acts upstream of the core pathway, either by a direct interaction between the two pathways or by biasing microtubule orientation and Fz/Dsh transport distally within the cell (reviewed in [34]). Nevertheless, the Ft-Ds system only influences trichome orientation over part of the wing, suggesting that other factors must also contribute to generating the global cue for the core pathway. These have been suggested to include growth, morphogenesis and mechanical tension [4,34,35].

## Molecular interactions of the core planar polarity pathway proteins

During pupal wing development, core pathway proteins are asymmetrically localised at the apico-lateral membrane, at the adherens zone. This polarisation is evident to some degree throughout development, but becomes more pronounced prior to trichome formation in the wing [36,37]. The establishment of core pathway planar polarity requires a first symmetry breaking step: the decision for Fz to accumulate distally and Stbm proximally around Fmi:Fmi homodimers at proximodistal cell boundaries (Figure 1C). In *fmi* mutant flies there is a reduction in Stbm and Pk at cell boundaries [22,23] and a loss of Fz, Dsh, and Dgo [17–20]. In *fz* mutant flies, Fmi is also mostly lost from the proximodistal cell boundaries and is observed at the apical cell membrane, suggesting that Fz is needed to stabilise Fmi homodimers [38]. Furthermore, Fmi appears to preferentially homodimerise when Fz is on only one side of the complex, suggesting that a Fz-Fmi:Fmi bridge is the backbone of the core pathway (Figure 1B) [38,39]. The cytosolic proteins and Stbm are required for the sorting of these asymmetric molecular bridges to a uniform orientation on proximodistal cell edges [17–20,22,23].

Beyond evidence that there is some polarised transport of Fz and Dsh distally on microtubule arrays [40,41], cellular mechanisms that sort the core proteins across the cell are not known. However, it has been reported that the core pathway proteins can self-organise. At cell boundaries in mosaic analysis experiments, *fz* mutant cells preferentially recruit Fz from their neighbours, whilst in the mutant cell Stbm localises to membranes apposing the wild-type neighbours, leading to a reversal of polarity on the distal side of the mosaic tissue [20,23,42,43]. This *de novo* planar polarity axis can extend across several cells suggesting an intracellular signal propagates away from the boundary cue [44].

Individual molecular interactions have been observed between many of the core pathway proteins. At least *in vitro* Stbm, Pk, Dsh, and Dgo can all interact with one another, while there also is evidence that Fmi may interact directly with Fz or Stbm, and Fz may interact with Stbm and Dsh (Table 2) [21–23,43,45–55]. Broadly speaking, these interactions are thought to underlie self-organisation, where complexes of the same orientation are stabilised, and complexes of opposite organisation are destabilised (Figure 1I–K). Feedback mechanisms driven by selective stabilisation/destabilisation of intercellular complexes could serve to amplify any small polarity bias generated by a global cue, to generate robust polarity [56–59].

**Table 2:** Physical interactions between the core planar polarity proteins

Protein 1	Protein 2	Method	References
Fmi	Fz	co-immunoprecipitation	[43]
Celsr1^1^	Vangl2^1^	co-immunoprecipitation	[52]
Fzd3^1^	Vangl2^1^	co-immunoprecipitation	[51]
Fzd5^1^	Dvl1^1^	PEPSCAN-based ELISA	[55]
Stbm Vangl2^1^	Stbm Vangl1^1^, Vangl2^1^	co-immunoprecipitation, GST pulldown, yeast two-hybrid	[46,54]
Stbm Vangl1^2^, Vangl2^2^	Pk Pk1^2^, Pk2^2^	co-immunoprecipitation, GST pulldown, mass spectrometry of immunoprecipitates	[23,46,53]
Stbm Vangl^3^^,^^4^ Vangl1^1^, Vangl2^1^	Dsh Dvl^3^^,^^4^ Dvl1^1^, Dvl2^1^, Dvl3^1^	co-immunoprecipitation, GST pulldown, yeast two-hybrid	[23,45,46,48]
Stbm	Dgo	GST pulldown	[21]
Pk	Pk	GST pulldown	[46]
Pk Pk^3^	Dsh Dvl^3^	GST pulldown	[22,47,49]
Pk	Dgo	GST pulldown	[21]
Dsh Dvl2^1^	Dgo Diversin^1^	co-immunoprecipitation, GST pulldown, yeast two-hybrid	[49,50]

See Table 1 for fly and vertebrate homologues. Interactions were between fly molecules unless otherwise stated.

1 Mouse;

2 human;

3 Xenopus;

4 zebrafish.

**Figure 2.**
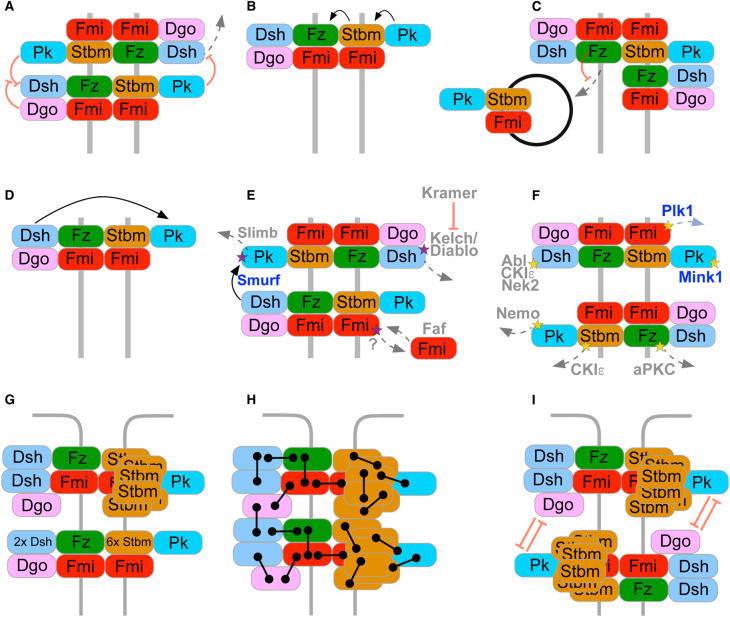
Mechanisms of planar cell polarity establishment. (**A**–**D**) Putative stabilising and destabilising interactions between the core proteins on two neighbouring cell membranes (grey solid lines). Black arrows indicate stabilising interactions and red inhibitory symbols indicate destabilising interactions. Dotted grey arrows indicate removal of proteins from the intercellular complex. (**A**) Pk and Dgo compete for Dsh binding. Pk binding to Dsh leads to destabilisation of Fz. (**B**) Pk acts through Stbm to stabilise Fz in the adjacent cell. (**C**) Pk promotes Fmi and Stbm endocytosis in the presence of Fz on the same membrane. (**D**) Dsh stabilises Pk in the adjacent cell. (**E**,**F**) Post-translational modifications of the core proteins. Purple stars indicate ubiquitination of a core protein (E) and yellow stars indicate phosphorylation (**F**). Modifying enzymes acting in *Drosophila* are in grey and those only known to act in vertebrates are in blue. (**G**) Stoichiometry of core pathway complexes. A homodimer of Fmi associates with one Fz, two Dsh and one Dgo molecule on one side of the complex and with six Stbm and one Pk molecule on the other. (**H**) Clustering of intercellular complexes in parallel arrays results in multiple stabilising interactions, and complexes undergo a phase transition to form stable puncta. (**I**) Outside of puncta, core protein complexes are in both orientations, and destabilising feedback interactions prevent clustering of complexes, leading to complexes that are less densely packed.

Some potential feedback mechanisms acting in core pathway self-organisation have been uncovered. Based on binding interactions, it was proposed that Dgo and Pk compete for binding to Dsh, with Dgo promoting Fz activity and Pk inhibiting it (Figure 2A) [22,49]. Consistent with this, more recent work has shown that Pk acts via Dsh to destabilise Fz in the same cell, and also acts to stabilise Fz via Stbm using trans-interactions in the neighbouring cell (Figure 2A,B) [60,61]. Furthermore, overexpression of Pk leads to the co-ordinated internalisation of Pk, Stbm and Fmi, and if Fz is forced to accumulate on particular cell boundaries in genetic mosaics, Pk promotes exclusion of Stbm from these cell boundaries (Figure 2C) [62]. In addition, Dsh binding to the Fz-Fmi:Fmi-Stbm complex results in the stabilisation of Pk in the adjacent cell [63]: if Dsh is acutely depleted in one cell, free Pk in the adjacent cell then destabilises Dsh-Fz in this cell, and thus leads to a propagating wave of core complex destabilisation (Figure 2D) [63].

In the pupal wing, core pathway proteins are observed along the length of the proximodistal junctional membranes in uneven abundance, and these localised concentrations are termed ‘puncta’. Fluorescence recovery after photobleaching (FRAP) experiments on live *in vivo* wing junctions have shown that core proteins in these puncta are more stable than in the non-puncta regions [64,65]. The proteins within these puncta are also highly asymmetrically localised compared with neighbouring non-puncta regions [62,64]. These data support the idea of mutual exclusion of unsorted complexes: antiparallel complexes are excluded from uniformly oriented puncta, while sorted complexes are mutually stabilised (Figure 1J,K).

Strutt and Gamage [65] directly measured the stoichiometry of core pathway proteins in these puncta, by tagging each protein individually with EGFP and comparing intensity in live pupal wings. When compared with immunolabelled Fmi, signal intensity of all six core proteins increased linearly with puncta size. This supports the idea that all puncta have the same composition. The stoichiometry in puncta was determined to be two Fmi (trans-homodimer) with approximately two Dsh, one Fz, and one Dgo on the distal membrane, and six Stbm with one Pk on the proximal membrane of the adjoining cell (Figure 2G). Interestingly, this stoichiometry is consistent between stable puncta and unstable non-puncta regions within the same cell.

Notably, the stoichiometry of the cytoplasmic proteins in puncta is dependent on their expression levels [65]. For example, if Dsh was overexpressed, more was found within both stable puncta and unstable non-puncta regions. However, the other complex components maintained their normal ratios. Likewise, when Dsh levels were halved, the ratio within stable and unstable regions halved for only Dsh [65]. In contrast, halving the expression of Fmi resulted in a halving of Fz recruitment into puncta, maintaining the 2 : 1 ratio of Fmi:Fz and supporting the asymmetric molecular bridge hypothesis (Figure 1B). This suggests that the cytosolic proteins form a dosage-dependent ‘cloud' around a fixed Fz-Fmi:Fmi backbone. The conclusion of this work is that core protein complexes within both stable puncta and unstable non-puncta coalesce via multivalent protein–protein interactions between the different proteins. Overall, it was suggested that when locally ordered complexes reach a critical density they undergo a phase transition from an unstable, fluid state, to a more solid stable state in puncta (Figure 2H,I) [65].

The Ft and Ds proteins are also organised into puncta, with FRAP experiments showing a more stable population in puncta [28]. Analogous to the core pathway proteins, puncta formation is dependent on formation of intercellular heterodimers between Ft and Ds. Furthermore, puncta stability is dependent on trans-binding affinity [28].

## Post-translational modifications influence protein–protein interactions and complex sorting

Post-translational modifications (PTMs) are ubiquitous in mediating feedback interactions in cell polarity, from symmetry breaking in yeast budding, to anteroposterior patterning of PAR proteins in *C. elegans* embryos and apicobasal polarity of epithelial cells [66–68]. The core pathway proteins have a complex network of PTMs that include ubiquitination and phosphorylation (Table 3). Ubiquitination of a protein can target it for proteasomal degradation, or can promote internalisation of transmembrane proteins that are then either recycled back to the plasma membrane or degraded in the lysosome [69]. Therefore, targeted ubiquitination of a core protein could promote destabilisation/removal of that protein from a complex and complex sorting. Phosphorylation can alter protein stability by priming proteins for ubiquitination, but it can also alter protein conformation and/or affinity for binding partners [70]; and thus could also regulate interactions between the core proteins and sorting of complexes. Interestingly, phosphorylation has been identified as a key modulator of phase transitions, and thus could regulate clustering of core protein complexes into puncta [71].

**Table 3:** Post-translational modifications of the core planar polarity proteins

Substrate	Enzyme	Candidate modification sites	References
Fmi^1^	Fat facets (Faf) deubiquitinase	-	[73]
Celsr1^2^	Polo-like kinase 1 (Plk1)^2^	14 S/T residues in the cytoplasmic domain of mouse Celsr1, including S2741, T2750, and S2752	[92]
Fz	atypical Protein Kinase C (aPKC)	S554, S560 of fly Fz	[95]
Stbm Vangl2^2^	Casein Kinase 1ε (CKIε) Casein Kinase 1δ/ε (CKIδ/ε)^2^	Two clusters of highly conserved S/T residues located in the N-terminal cytoplasmic domain (S5–17, ST113–122 of fly Dsh; S5–17, ST76–84 of mouse Vangl2)	[61,88–91]
Pk	Farnesyl-diphosphate farnesyl transferase (FNTA, FNTB)	C-terminal CaaX motif	[75]
Pk Pk2^2^	Cullin1-SkpA-Slimb Skp1-Cullin1-F-Box^2^ ubiquitin ligase	-	[62,75,76]
Pk1^2^	Smurf1^2^, Smurf2^2^ ubiquitin ligases	-	[77,78]
Pk	Nemo kinase	4 potential MAPK phosphorylation sites in the middle region	[98]
Pk1	Ste20 kinase Mink1	T370 of *Xenopus* Pk1	[53]
Dsh Dvl^3^	Cullin3-Diablo/Kelch Cullin3-KLHL12^3^ ubiquitin ligase	-	[72,73]
Dsh	Abelson tyrosine kinase (Abl)	Y473 of fly Dsh	[79,82]
Dsh	Nek2 kinase	multiple sites	[80,81]
Dsh Dvl^2^^,^^3^^,^^4^	Casein Kinase 1ε (CKIε) Casein Kinase 1δ/ε (CKIδ/ε)^2^^,^^3^^,^^4^	S236 of fly Dsh	[61,83–87]

See Table 1 for fly and vertebrate homologues. Modifications were of fly molecules unless otherwise stated.

1 Deduced target, but no direct modification observed;

2 Mouse;

3 human;

4 Xenopus.

Ubiquitination of *Drosophila* Dsh via a Cullin3-Diablo/Kelch ubiquitin ligase complex results in reduced Dsh at cell junctions and its targeting for degradation [72,73]. Dsh ubiquitination is antagonised by the phosphoinositide binding protein Kramer/PLEKHA4, which in vertebrates sequesters the Kelch homologue KLHL12 (Figure 2E) [74]. There is also evidence of ubiquitination promoting internalisation of Fmi, as the deubiquitinase Fat facets (Faf) promotes the recycling of internalised Fmi to the junctional membrane (Figure 2E) [73]. Furthermore, farnesylation of Pk promotes its recruitment into the proximal complex by Stbm [75], while Stbm also promotes degradation of excess Pk via a Cullin1–SkpA–Supernumerary limbs (Slimb) ubiquitin ligase complex (Figure 2E) [62,75,76]. In vertebrates, the HECT ubiquitin ligases Smurf1 and Smurf2 promote proteasomal degradation of Pk [77]. Interestingly, Smurfs also interact specifically with phosphorylated Dvl (the vertebrate Dsh homologue) [77], and Dvl promotes Smurf activity [78]. This leads to a model in which phosphorylated Dsh recruits Smurf, which then ubiquitinates Pk and destabilises complexes of the opposite orientation (Figure 2E).

The abundance of evidence for PTM control of planar polarity comes from assays of protein phosphorylation. In particular, Dsh hyperphosphorylation correlates with its recruitment to junctions by Fz [17,19], and analysis of putative phosphorylation sites in Dsh has revealed multiple redundant phosphorylation sites [79]. Phosphorylation of Dsh by the Abelson tyrosine kinase (Abl), Nek2 and Casein kinase Iε (CKIε, or Discs overgrown [Dco] in flies) has been implicated in regulation of planar polarity (Figure 2F). Nek2 phosphorylates Dsh at multiple sites [80] and loss of Nek2 leads to accumulation of Dsh in intracellular puncta, and reduced core protein asymmetry [81]. Abl phosphorylates Dsh *in vitro* at Tyr473, and loss of Abl disrupts photoreceptor specification and polarity [82]. Finally, CKIε promotes Dsh phosphorylation *in vitro* and *in vivo*, and loss of CKIε abolishes core protein asymmetry [61,83–88].

In addition to phosphorylating Dsh, CKIε also phosphorylates the N-terminus of Stbm, and mutation of the Stbm phosphorylation sites disrupts planar polarity in both flies and vertebrates [61,88–91]. Interestingly, in *Drosophila* mutation of the CKIε phosphorylation sites in Stbm leads to an increase in Stbm stability, while mutation of putative CKIε phosphorylation sites in Dsh decreases its stability (Figure 2F) [61]. This dual action of CKIε would therefore enhance removal of Stbm from complexes and stabilise Dsh in complexes, and thus promote sorting of proximal and distal complex components to opposite cell ends.

In vertebrate skin, the Fmi homologue Celsr1 is phosphorylated on a dileucine endocytic motif by Polo-like kinase 1 (Plk1) [92]. Interestingly, Plk1 is a mitotic kinase and thus promotes internalisation of Celsr1 specifically in mitosis (Figure 2F). Failure of this internalisation perturbs skin planar polarity [92,93]. Internalisation was suggested to allow core proteins to be redistributed equally between the two daughter cells, and also to protect mitotic cells against aberrant signalling while they are rounded up.

Three further kinases, atypical PKC (aPKC), Nemo and Misshapen (Msn) have also been implicated in regulating planar polarity in the *Drosophila* eye (Figure 2F) [94–96]. aPKC can phosphorylate the cytoplasmic tail of Fz, which is thought to inhibit Fz function. This activity of aPKC on Fz is antagonised by the apicobasal polarity protein Bazooka/Par-3 [95]. Bazooka also interacts with Fmi, raising interesting questions regarding cross-talk between apicobasal and planar polarity [97]. Nemo is thought to act in two ways in eye planar polarity: firstly, a direct effect via Pk [98] and secondly by regulating downstream effectors that regulate ommatidial cluster rotation [99]. Pk phosphorylation by Nemo is thought to promote the targeted proteasomal degradation of Pk from planar polarity complexes via Cullin1–SkpA–Slimb (see above) [98]. Finally, in vertebrates Pk phosphorylation by the Msn homologue Mink1 promotes Pk trafficking to the plasma membrane and clustering with the vertebrate Stbm homologue Vangl2 [53].

## Future directions

It is interesting to note that despite the long list of known PTMs that affect core protein activity, only in the case of Smurf binding to phosphorylated Dsh has a specific regulatory mechanism been uncovered that implicates a PTM in driving core complex sorting within clusters. Nevertheless, it seems likely that spatiotemporal regulation of PTMs will be a critical mechanism in planar polarity establishment.

Integral to models of core protein sorting via feedback interactions is the idea that complexes in the same orientation are stabilised and those of the opposite orientation are destabilised (Figure 1J). To understand stabilising/destabilising interactions it will be necessary to identify the role of specific PTMs: in particular, how each PTM affects the stability of core proteins at junctions. It will also be important to determine whether and how specific PTMs are regulated: for example if one core protein locally promotes the modification of another protein and this alters its stability then this would constitute evidence for a feedback mechanism.

Moreover, understanding the complex roles of multiple PTMs will require precise spatiotemporal manipulations of protein activity, in order to distinguish fast acting direct effects from more long term consequences. This will require the development of new methods for acutely activating or removing activity. Finally, we anticipate that advances in microscopy — for instance single molecule imaging — that allow more detailed visualisation of core protein clustering at cell junctions, will be key in understanding the effects of specific PTMs on core protein organisation and sorting.

Such studies will be an important and exciting way forward in unravelling molecular mechanisms of core planar polarity pathway function.

## Perspectives

- Planar polarity allows cells and groups of cells to exhibit polarised behaviours that are co-ordinated with their neighbours and the tissue axes. Such coordination of cell behaviours is vital for correct tissue morphogenesis and function.
- Co-ordinated planar polarisation involves the formation of heterophilic intercellular complexes that become asymmetrically localised to opposite cell edges. Self-organising processes involving stabilising and destabilising feedback interactions between intercellular complexes leads to clustering of complexes of the same orientation in puncta. PTMs most likely play a key role in controlling the spatiotemporal dynamics of planar polarity protein interactions.
- Further research is required to elucidate the cellular mechanisms that sort the core proteins across the cell, and how the feedback interactions between the core planar polarity proteins are regulated. The control of PTMs is important in core protein asymmetry, however, the mechanisms controlling PTM activity are poorly understood. Better methods for mapping PTMs and PTM enzyme activity in time and space will be essential for understanding pathway function.

## Abbreviations

FRAP: Fluorescence recovery after photobleaching
PCP: planar cell polarity
PTMs: Post-translational modifications

## References

[1] Hale R. and Strutt D. (2015) Conservation of planar polarity pathway function across the animal kingdom. Annu. Rev. Genet. 49, 529–551 10.1146/annurev-genet-112414-055224 26360326

[2] Goodrich L.V. and Strutt D. (2011) Principles of planar polarity in animal development. Development 138, 1877–1892 10.1242/dev.054080 21521735 PMC3082295

[3] Devenport D. (2014) The cell biology of planar cell polarity. J. Cell Biol. 207, 171–179 10.1083/jcb.201408039 25349257 PMC4210441

[4] Butler M.T. and Wallingford J.B. (2017) Planar cell polarity in development and disease. Nat. Rev. Mol. Cell. Biol. 18, 375–388 10.1038/nrm.2017.11 28293032 PMC5826606

[5] Gong Y., Mo C. and Fraser S.E. (2004) Planar cell polarity signalling controls cell division orientation during zebrafish gastrulation. Nature 430, 689–693 10.1038/nature02796 15254551

[6] Trichas G., Smith A.M., White N., Wilkins V., Watanabe T., Moore A. et al. (2012) Multi-cellular rosettes in the mouse visceral endoderm facilitate the ordered migration of anterior visceral endoderm cells. PLoS Biol. 10, e1001256 10.1371/journal.pbio.1001256 22346733 PMC3274502

[7] Wang M., Marco P., Capra V. and Kibar Z. (2019) Update on the role of the non-canonical Wnt/planar cell polarity pathway in neural tube defects. Cells 8, 1198 10.3390/cells8101198 PMC6829399 31590237

[8] Chen H. and Cheng C.Y. (2016) Planar cell polarity (PCP) proteins and spermatogenesis. Semin. Cell Dev. Biol. 59, 99–109 10.1016/j.semcdb.2016.04.010 27108805 PMC5071175

[9] Tower-Gilchrist C., Zlatic S.A., Yu D., Chang Q., Wu H., Lin X. et al. (2019) Adaptor protein-3 complex is required for Vangl2 trafficking and planar cell polarity of the inner ear. Mol. Biol. Cell 30, 2422–2434 10.1091/mbc.E16-08-0592 31268833 PMC6741063

[10] Henderson D.J. and Chaudhry B. (2011) Getting to the heart of planar cell polarity signaling. Birth Defects Res. A Clin. Mol. Teratol. 91, 460–467 10.1002/bdra.20792 21538810

[11] Ambegaonkar A.A., Pan G., Mani M., Feng Y. and Irvine K.D. (2012) Propagation of Dachsous-Fat planar cell polarity. Curr. Biol. 22, 1302–1308 10.1016/j.cub.2012.05.049 22727698 PMC3418676

[12] Bosveld F., Bonnet I., Guirao B., Tlili S., Wang Z., Petitalot A. et al. (2012) Mechanical control of morphogenesis by Fat/Dachsous/Four-jointed planar cell polarity pathway. Science 336, 724–727 10.1126/science.1221071 22499807

[13] Brittle A., Thomas C. and Strutt D. (2012) Planar polarity specification through asymmetric subcellular localization of Fat and dachsous. Curr. Biol. 22, 907–914 10.1016/j.cub.2012.03.053 22503504 PMC3362735

[14] Ma D., Yang C.H., McNeill H., Simon M.A. and Axelrod J.D. (2003) Fidelity in planar cell polarity signalling. Nature 421, 543–547 10.1038/nature01366 12540853

[15] Matakatsu H. and Blair S.S. (2004) Interactions between Fat and Dachsous and the regulation of planar cell polarity in the *drosophila* wing. Development 131, 3785–3794 10.1242/dev.01254 15240556

[16] Usui T., Shima Y., Shimada Y., Hirano S., Burgess R.W., Schwarz T.L. et al. (1999) Flamingo, a seven-pass transmembrane cadherin, regulates planar cell polarity under the control of frizzled. Cell 98, 585–595 10.1016/S0092-8674(00)80046-X 10490098

[17] Axelrod J.D. (2001) Unipolar membrane association of dishevelled mediates frizzled planar cell polarity signaling. Genes Dev. 15, 1182–1187 10.1101/gad.890501 11358862 PMC313798

[18] Feiguin F., Hannus M., Mlodzik M. and Eaton S. (2001) The ankyrin repeat protein Diego mediates Frizzled-dependent planar polarization. Dev. Cell 1, 93–101 10.1016/S1534-5807(01)00010-7 11703927

[19] Shimada Y., Usui T., Yanagawa S., Takeichi M. and Uemura T. (2001) Asymmetric colocalization of Flamingo, a seven-pass transmembrane cadherin, and dishevelled in planar cell polarization. Curr. Biol. 11, 859–863 10.1016/S0960-9822(01)00233-0 11516647

[20] Strutt D.I. (2001) Asymmetric localization of Frizzled and the establishment of cell polarity in the *Drosophila* wing. Mol. Cell 7, 367–375 10.1016/S1097-2765(01)00184-8 11239465

[21] Das G., Jenny A., Klein T.J., Eaton S. and Mlodzik M. (2004) Diego interacts with Prickle and Strabismus/Van Gogh to localize planar cell polarity complexes. Development 131, 4467–4476 10.1242/dev.01317 15306567

[22] Tree D.R., Shulman J.M., Rousset R., Scott M.P., Gubb D. and Axelrod J.D. (2002) Prickle mediates feedback amplification to generate asymmetric planar cell polarity signaling. Cell 109, 371–381 10.1016/S0092-8674(02)00715-8 12015986

[23] Bastock R., Strutt H. and Strutt D. (2003) Strabismus is asymmetrically localised and binds to Prickle and Dishevelled during *Drosophila* planar polarity patterning. Development 130, 3007–3014 10.1242/dev.00526 12756182

[24] Zeidler M.P., Perrimon N. and Strutt D.I. (2000) Multiple rôles for *four-jointed* in planar polarity and limb patterning. Dev. Biol. 228, 181–196 10.1006/dbio.2000.9940 11112323

[25] Strutt H., Mundy J., Hofstra K. and Strutt D. (2004) Cleavage and secretion is not required for four-jointed function in *Drosophila* patterning. Development 131, 881–890 10.1242/dev.00996 14757640

[26] Brittle A.L., Repiso A., Casal J., Lawrence P.A. and Strutt D. (2010) Four-Jointed modulates growth and planar polarity by reducing the affinity of Dachsous for Fat. Curr. Biol. 20, 803–810 10.1016/j.cub.2010.03.056 20434337 PMC2958304

[27] Simon M.A., Xu A., Ishikawa H.O. and Irvine K.D. (2010) Modulation of Fat:Dachsous binding by the cadherin domain kinase four-Jointed. Curr. Biol. 20, 811–817 10.1016/j.cub.2010.04.016 20434335 PMC2884055

[28] Hale R., Brittle A.L., Fisher K.H., Monk N.A. and Strutt D. (2015) Cellular interpretation of the long-range gradient of four-jointed activity in the *Drosophila* wing. eLife 4, e05789 10.7554/eLife.05789 PMC4338440 25707557

[29] Bhanot P., Brink M., Samos C.H., Hsieh J.-C., Wang Y., Macke J.P. et al. (1996) A new member of the *frizzled* family from *Drosophila* functions as a wingless receptor. Nature 382, 225–230 10.1038/382225a0 8717036

[30] Strigini M. and Cohen S.M. (1999) Formation of morphogen gradients in the *Drosophila* wing. Semin. Cell Dev. Biol. 10, 335–344 10.1006/scdb.1999.0293 10441548

[31] Gieseler K., Wilder E., Mariol M.C., Buratovitch M., Berenger H., Graba Y. et al. (2001) DWnt4 and wingless elicit similar cellular responses during imaginal development. Dev. Biol. 232, 339–350 10.1006/dbio.2001.0184 11401396

[32] Janson K., Cohen E.D. and Wilder E.L. (2001) Expression of DWnt6, DWnt10, and DFz4 during *Drosophila* development. Mech. Dev. 103, 117–120 10.1016/S0925-4773(01)00323-9 11335117

[33] Wu J., Roman A.C., Carvajal-Gonzalez J.M. and Mlodzik M. (2013) Wg and Wnt4 provide long-range directional input to planar cell polarity orientation in *drosophila*. Nat. Cell Biol. 15, 1045–1055 10.1038/ncb2806 23912125 PMC3762953

[34] Aw W.Y. and Devenport D. (2016) Planar cell polarity: global inputs establishing cellular asymmetry. Curr. Opin. Cell Biol. 44, 110–116 10.1016/j.ceb.2016.08.002 27576155 PMC5326696

[35] Sagner A., Merkel M., Aigouy B., Gaebel J., Brankatschk M., Julicher F. et al. (2012) Establishment of global patterns of planar polarity during growth of the *drosophila* wing epithelium. Curr. Biol. 22, 1296–1301 10.1016/j.cub.2012.04.066 22727699

[36] Classen A.K., Anderson K.I., Marois E. and Eaton S. (2005) Hexagonal packing of *drosophila* wing epithelial cells by the planar cell polarity pathway. Dev. Cell 9, 805–817 10.1016/j.devcel.2005.10.016 16326392

[37] Aigouy B., Farhadifar R., Staple D.B., Sagner A., Röper J.-C., Julicher F. et al. (2010) Cell flow reorients the axis of planar polarity in the wing epithelium of *Drosophila*. Cell 142, 773–786 10.1016/j.cell.2010.07.042 20813263

[38] Strutt H. and Strutt D. (2008) Differential stability of Flamingo protein complexes underlies the establishment of planar polarity. Curr. Biol. 18, 1555–1564 10.1016/j.cub.2008.08.063 18804371 PMC2593845

[39] Struhl G., Casal J. and Lawrence P.A. (2012) Dissecting the molecular bridges that mediate the function of Frizzled in planar cell polarity. Development 139, 3665–3674 10.1242/dev.083550 22949620 PMC3436116

[40] Shimada Y., Yonemura S., Ohkura H., Strutt D. and Uemura T. (2006) Polarized transport of Frizzled along the planar microtubule arrays in *Drosophila* wing epithelium. Dev. Cell 10, 209–222 10.1016/j.devcel.2005.11.016 16459300

[41] Matis M., Russler-Germain D.A., Hu Q., Tomlin C.J. and Axelrod J.D. (2014) Microtubules provide directional information for core PCP function. eLife 3, e02893 10.7554/eLife.02893 25124458 PMC4151085

[42] Strutt D. and Strutt H. (2007) Differential activities of the core planar polarity proteins during *Drosophila* wing patterning. Dev. Biol. 302, 181–194 10.1016/j.ydbio.2006.09.026 17045581 PMC2082130

[43] Chen W.S., Antic D., Matis M., Logan C.Y., Povelones M., Anderson G.A. et al. (2008) Asymmetric homotypic interactions of the atypical cadherin Flamingo mediate intercellular polarity signaling. Cell 133, 1093–1105 10.1016/j.cell.2008.04.048 18555784 PMC2446404

[44] Vinson C.R. and Adler P.N. (1987) Directional non-cell autonomy and the transmission of polarity information by the *frizzled* gene of *Drosophila*. Nature 329, 549–551 10.1038/329549a0 3116434

[45] Park M. and Moon R.T. (2002) The planar cell-polarity gene *stbm* regulates cell behaviour and cell fate in vertebrate embryos. Nat. Cell Biol. 4, 20–25 10.1038/ncb716 11780127

[46] Jenny A., Darken R.S., Wilson P.A. and Mlodzik M. (2003) Prickle and strabismus form a functional complex to generate a correct axis during planar cell polarity signaling. EMBO J. 22, 4409–4420 10.1093/emboj/cdg424 12941693 PMC202366

[47] Takeuchi M., Nakabayashi J., Sakaguchi T., Yamamoto T.S., Takahashi H., Takeda H. et al. (2003) The *prickle*-related gene in vertebrates is essential for gastrulation cell movements. Curr. Biol. 13, 674–679 10.1016/S0960-9822(03)00245-8 12699625

[48] Torban E., Wang H.J., Groulx N. and Gros P. (2004) Independent mutations in mouse Vangl2 that cause neural tube defects in looptail mice impair interaction with members of the dishevelled family. J. Biol. Chem. 279, 52703–52713 10.1074/jbc.M408675200 15456783

[49] Jenny A., Reynolds-Kenneally J., Das G., Burnett M. and Mlodzik M. (2005) Diego and Prickle regulate Frizzled planar cell polarity signalling by competing for dishevelled binding. Nat. Cell Biol. 7, 691–697 10.1038/ncb1271 15937478

[50] Moeller H., Jenny A., Schaeffer H.J., Schwarz-Romond T., Mlodzik M., Hammerschmidt M. et al. (2006) Diversin regulates heart formation and gastrulation movements in development. Proc. Natl Acad. Sci. U.S.A. 103, 15900–15905 10.1073/pnas.0603808103 17032765 PMC1635100

[51] Montcouquiol M., Sans N., Huss D., Kach J., Dickman J.D., Forge A. et al. (2006) Asymmetric localization of Vangl2 and Fz3 indicate novel mechanisms for planar cell polarity in mammals. J. Neurosci. 26, 5265–5275 10.1523/JNEUROSCI.4680-05.2006 16687519 PMC6674235

[52] Devenport D. and Fuchs E. (2008) Planar polarization in embryonic epidermis orchestrates global asymmetric morphogenesis of hair follicles. Nat. Cell Biol. 10, 1257–1268 10.1038/ncb1784 18849982 PMC2607065

[53] Daulat A.M., Luu O., Sing A., Zhang L., Wrana J.L., McNeill H. et al. (2012) Mink1 regulates beta-catenin-independent Wnt signaling via Prickle phosphorylation. Mol. Cell. Biol. 32, 173–185 10.1128/MCB.06320-11 22037766 PMC3255712

[54] Belotti E., Puvirajesinghe T.M., Audebert S., Baudelet E., Camoin L., Pierres M. et al. (2012) Molecular characterisation of endogenous Vangl2/Vangl1 heteromeric protein complexes. PLoS ONE 7, e46213 10.1371/journal.pone.0046213 23029439 PMC3460870

[55] Tauriello D.V., Jordens I., Kirchner K., Slootstra J.W., Kruitwagen T., Bouwman B.A. et al. (2012) Wnt/beta-catenin signaling requires interaction of the dishevelled DEP domain and C terminus with a discontinuous motif in Frizzled. Proc. Natl Acad. Sci. U.S.A. 109, E812–E820 10.1073/pnas.1114802109 22411803 PMC3325702

[56] Amonlirdviman K., Khare N.A., Tree D.R., Chen W.S., Axelrod J.D. and Tomlin C.J. (2005) Mathematical modeling of planar cell polarity to understand domineering nonautonomy. Science 307, 423–426 10.1126/science.1105471 15662015

[57] Le Garrec J.F., Lopez P. and Kerszberg M. (2006) Establishment and maintenance of planar epithelial cell polarity by asymmetric cadherin bridges: a computer model. Dev. Dyn. 235, 235–246 10.1002/dvdy.20617 16258926

[58] Burak Y. and Shraiman B.I. (2009) Order and stochastic dynamics in *Drosophila* planar cell polarity. PLoS Comput. Biol. 5, e1000628 10.1371/journal.pcbi.1000628 20041171 PMC2791803

[59] Schamberg S., Houston P., Monk N.A. and Owen M.R. (2010) Modelling and analysis of planar cell polarity. Bull. Math. Biol. 72, 645–680 10.1007/s11538-009-9464-0 20107923

[60] Warrington S.J., Strutt H., Fisher K.H. and Strutt D. (2017) A dual function for Prickle in regulating Frizzled stability during feedback-dependent amplification of planar polarity. Curr. Biol. 27, 2784–2797 10.1016/j.cub.2017.08.016 28918952 PMC5628951

[61] Strutt H., Gamage J. and Strutt D. (2019) Reciprocal action of casein kinase I epsilon on core planar polarity proteins regulates clustering and asymmetric localisation. eLife 8, e45107 10.7554/eLife.45107 31090542 PMC6542583

[62] Cho B., Pierre-Louis G., Sagner A., Eaton S. and Axelrod J.D. (2015) Clustering and negative feedback by endocytosis in planar cell polarity signaling is modulated by ubiquitinylation of prickle. PLoS Genet. 11, e1005259 10.1371/journal.pgen.1005259 25996914 PMC4440771

[63] Ressurreicao M., Warrington S. and Strutt D. (2018) Rapid disruption of dishevelled activity uncovers an intercellular role in maintenance of Prickle in core planar polarity protein complexes. Cell Rep. 25, 1415–1424 10.1016/j.celrep.2018.10.039 30403998 PMC6231328

[64] Strutt H., Warrington S.J. and Strutt D. (2011) Dynamics of core planar polarity protein turnover and stable assembly into discrete membrane subdomains. Dev. Cell 20, 511–525 10.1016/j.devcel.2011.03.018 21497763 PMC3094756

[65] Strutt H., Gamage J. and Strutt D. (2016) Robust asymmetric localization of planar polarity proteins is associated with organization into signalosome-like domains of variable stoichiometry. Cell. Rep. 17, 2660–2671 10.1016/j.celrep.2016.11.021 27926869 PMC5177602

[66] Chiou J.G., Balasubramanian M.K. and Lew D.J. (2017) Cell polarity in yeast. Annu. Rev. Cell Dev. Biol. 33, 77–101 10.1146/annurev-cellbio-100616-060856 28783960 PMC5944360

[67] Lang C.F. and Munro E. (2017) The PAR proteins: from molecular circuits to dynamic self-stabilizing cell polarity. Development 144, 3405–3416 10.1242/dev.139063 28974638 PMC5665476

[68] St Johnston D. (2018) Establishing and transducing cell polarity: common themes and variations. Curr. Opin. Cell Biol. 51, 33–41 10.1016/j.ceb.2017.10.007 29153703

[69] Clague M.J. and Urbe S. (2010) Ubiquitin: same molecule, different degradation pathways. Cell 143, 682–685 10.1016/j.cell.2010.11.012 21111229

[70] Hunter T. (2007) The age of crosstalk: phosphorylation, ubiquitination, and beyond. Mol. Cell 28, 730–738 10.1016/j.molcel.2007.11.019 18082598

[71] Snead W.T. and Gladfelter A.S. (2019) The control centers of biomolecular phase separation: how membrane surfaces, PTMs, and active processes regulate condensation. Mol. Cell 76, 295–305 10.1016/j.molcel.2019.09.016 31604601 PMC7173186

[72] Angers S., Thorpe C.J., Biechele T.L., Goldenberg S.J., Zheng N., MacCoss M.J. et al. (2006) The KLHL12-Cullin-3 ubiquitin ligase negatively regulates the Wnt-beta-catenin pathway by targeting dishevelled for degradation. Nat. Cell Biol. 8, 348–357 10.1038/ncb1381 16547521

[73] Strutt H., Searle E., Thomas-Macarthur V., Brookfield R. and Strutt D. (2013) A Cul-3-BTB ubiquitylation pathway regulates junctional levels and asymmetry of core planar polarity proteins. Development 140, 1693–1702 10.1242/dev.089656 23487316 PMC3621487

[74] Shah A. S., Batrouni A.G., Kim D., Punyala A., Cao W., Han C. et al. (2019) PLEKHA4/kramer attenuates dishevelled ubiquitination to modulate Wnt and planar cell polarity signaling. Cell Rep. 27, 2157–2170 10.1016/j.celrep.2019.04.060 31091453 PMC6594551

[75] Strutt H., Thomas-MacArthur V. and Strutt D. (2013) Strabismus promotes recruitment and degradation of farnesylated prickle in *Drosophila melanogaster* planar polarity specification. PLoS Genet. 9, e1003654 10.1371/journal.pgen.1003654 23874239 PMC3715439

[76] Nagaoka T., Furuse M., Ohtsuka T., Tsuchida K. and Kishi M. (2019) Vangl2 interaction plays a role in the proteasomal degradation of Prickle2. Sci. Rep. 9, 2912 10.1038/s41598-019-39642-z 30814664 PMC6393536

[77] Narimatsu M., Bose R., Pye M., Zhang L., Miller B., Ching P. et al. (2009) Regulation of planar cell polarity by smurf ubiquitin ligases. Cell 137, 295–307 10.1016/j.cell.2009.02.025 19379695

[78] Bernatik O., Paclikova P., Sri Ganji R. and Bryja V. (2020) Activity of Smurf2 ubiquitin ligase Is regulated by the Wnt pathway protein dishevelled. Cells 9, 1147 10.3390/cells9051147 PMC7290506 32392721

[79] Yanfeng W.A., Berhane H., Mola M., Singh J., Jenny A. and Mlodzik M. (2011) Functional dissection of phosphorylation of disheveled in *drosophila*. Dev. Biol. 360, 132–142 10.1016/j.ydbio.2011.09.017 21963539 PMC3221411

[80] Schertel C., Huang D., Bjorklund M., Bischof J., Yin D., Li R. et al. (2013) Systematic screening of a *Drosophila* ORF library in vivo uncovers Wnt/Wg pathway components. Dev. Cell 25, 207–219 10.1016/j.devcel.2013.02.019 23583758

[81] Weber U. and Mlodzik M. (2017) APC/c(Fzr/Cdh1)-dependent regulation of planar cell polarity establishment via Nek2 kinase acting on dishevelled. Dev. Cell 40, 53–66 10.1016/j.devcel.2016.12.006 28041906 PMC5225046

[82] Singh J., Yanfeng W.A., Grumolato L., Aaronson S.A. and Mlodzik M. (2010) Abelson family kinases regulate Frizzled planar cell polarity signaling via Dsh phosphorylation. Genes Dev. 24, 2157–2168 10.1101/gad.1961010 20837657 PMC2947768

[83] Peters J.M., McKay R.M., McKay J.P. and Graff J.M. (1999) Casein kinase I transduces Wnt signals. Nature 401, 345–350 10.1038/43830 10517632

[84] Sakanaka C., Leong P., Xu L., Harrison S.D. and Williams L.T. (1999) Casein kinase Iε in the Wnt pathway: Regulation of β-catenin function. Proc. Natl Acad. Sci. U.S.A. 96, 12548–12552 10.1073/pnas.96.22.12548 10535959 PMC22983

[85] Cong F., Schweizer L. and Varmus H. (2004) Casein kinase Iε modulates the signaling specificities of dishevelled. Mol. Cell. Biol. 24, 2000–2011 10.1128/MCB.24.5.2000-2011.2004 14966280 PMC350543

[86] Klein T.J., Jenny A., Djiane A. and Mlodzik M. (2006) CKIepsilon/discs overgrown promotes both Wnt-Fz/beta-catenin and Fz/PCP signaling in *drosophila*. Curr. Biol. 16, 1337–1343 10.1016/j.cub.2006.06.030 16824922

[87] Strutt H., Price M.A. and Strutt D. (2006) Planar polarity is positively regulated by casein kinase iepsilon in *drosophila*. Curr. Biol. 16, 1329–1336 10.1016/j.cub.2006.04.041 16824921

[88] Kelly L.K., Wu J., Yanfeng W.A. and Mlodzik M. (2016) Frizzled-induced Van Gogh phosphorylation by CK1epsilon promotes asymmetric localization of core PCP factors in *Drosophila*. Cell Rep. 16, 344–356 10.1016/j.celrep.2016.06.010 27346358 PMC4945453

[89] Gao B., Song H., Bishop K., Elliot G., Garrett L., English M.A. et al. (2011) Wnt signaling gradients establish planar cell polarity by inducing Vangl2 phosphorylation through Ror2. Dev. Cell 20, 163–176 10.1016/j.devcel.2011.01.001 21316585 PMC3062198

[90] Ossipova O., Kim K. and Sokol S.Y. (2015) Planar polarization of Vangl2 in the vertebrate neural plate is controlled by Wnt and Myosin II signaling. Biol. Open. 4, 722–730 10.1242/bio.201511676 25910938 PMC4467192

[91] Yang W., Garrett L., Feng D., Elliott G., Liu X., Wang N. et al. (2017) Wnt-induced Vangl2 phosphorylation is dose-dependently required for planar cell polarity in mammalian development. Cell Res. 27, 1466–1484 10.1038/cr.2017.127 29056748 PMC5717403

[92] Shrestha R., Little K.A., Tamayo J.V., Li W., Perlman D.H. and Devenport D. (2015) Mitotic control of planar cell polarity by Polo-like kinase 1. Dev. Cell 33, 522–534 10.1016/j.devcel.2015.03.024 26004507 PMC4464975

[93] Devenport D., Oristian D., Heller E. and Fuchs E. (2011) Mitotic internalization of planar cell polarity proteins preserves tissue polarity. Nat. Cell Biol. 13, 893–902 10.1038/ncb2284 21743464 PMC3149741

[94] Choi K.W. and Benzer S. (1994) Rotation of photoreceptor clusters in the developing *Drosophila* eye requires the *nemo* gene. Cell 78, 125–136 10.1016/0092-8674(94)90579-7 8033204

[95] Djiane A. and Mlodzik M S.Y. (2005) The apical determinants aPKC and dPatj regulate Frizzled-dependent planar cell polarity in the *Drosophila* eye. Cell 121, 621–631 10.1016/j.cell.2005.03.014 15907474

[96] Paricio N., Feiguin F., Boutros M., Eaton S. and Mlodzik M. (1999) The Drosophila STE20-like kinase misshapen is required downstream of the Frizzled receptor in planar polarity signaling. EMBO J. 18, 4669–4678 10.1093/emboj/18.17.4669 10469646 PMC1171540

[97] Wasserscheid I., Thomas U. and Knust E. (2007) Isoform-specific interaction of Flamingo/Starry Night with excess Bazooka affects planar cell polarity in the *Drosophila* wing. Dev. Dyn. 236, 1064–1071 10.1002/dvdy.21089 17304516

[98] Collu G.M., Jenny A., Gaengel K., Mirkovic I., Chin M.L., Weber U. et al. (2018) Prickle is phosphorylated by Nemo and targeted for degradation to maintain Prickle/Spiny-legs isoform balance during planar cell polarity establishment. PLoS Genet. 14, e1007391 10.1371/journal.pgen.1007391 29758044 PMC5967807

[99] Mirkovic I., Gault W.J., Rahnama M., Jenny A., Gaengel K., Bessette D. et al. (2011) Nemo kinase phosphorylates beta-catenin to promote ommatidial rotation and connects core PCP factors to E-cadherin-beta-catenin. Nat. Struct. Mol. Biol. 18, 665–672 10.1038/nsmb.2049 21552260 PMC3109122

